# Human amnion‐derived mesenchymal stem cells promote osteogenesis of human bone marrow mesenchymal stem cells against glucolipotoxicity

**DOI:** 10.1002/2211-5463.12547

**Published:** 2018-11-26

**Authors:** Yifeng Bian, Xiaojie Ma, Ruixia Wang, Hua Yuan, Ning Chen, Yifei Du

**Affiliations:** ^1^ Jiangsu Key Laboratory of Oral Diseases Nanjing Medical University China; ^2^ Department of Dental Implant Affiliated Hospital of Stomatology Nanjing Medical University China; ^3^ Department of Oral and Maxillofacial Surgery Affiliated Hospital of Stomatology Nanjing Medical University China

**Keywords:** glucolipotoxicity, human amnion‐derived mesenchymal stem cells, human bone marrow mesenchymal stem cells, osteogenic differentiation

## Abstract

Epidemiological evidence suggests that diabetes mellitus (DM) is an important factor in promoting periodontitis. It not only affects the attachment of connective tissue but also causes loss of alveolar bone. Hence, there is an urgent need to find an effective treatment for DM‐induced bone deficiency. This study aimed to investigate the effects of human amniotic mesenchymal stem cells (HAMSCs) on the proliferation and osteogenic differentiation of DM‐induced human bone marrow mesenchymal stem cells (HBMSCs). High glucose and palmitic acid (GP) were used to mimic DM‐induced glucolipotoxicity. The proliferation levels were measured using flow cytometry. Alkaline phosphatase activity substrate assays, Alizarin red S staining, and western blotting were used to investigate osteogenic differentiation. Oxidative stress was measured by assaying the levels of reactive oxygen species. This study found that glucolipotoxicity caused by GP remarkably inhibited cell proliferation and osteogenesis, and upregulated the oxidative stress level in HBMSCs. However, HAMSCs attenuated HBMSC dysfunction through antioxidant activity by influencing p38 mitogen‐activated protein kinase and vascular endothelial growth factor secretion. In conclusion, our findings indicate that HAMSCs might be suitable for treating DM‐mediated bone deficiency.

**Abbreviations**
ALP: alkaline phosphatase
DCF: 2,7‐dichlorodihydrofluorescein
DCFH‐DA: 2,7‐dichlorodihydrofluorescein diacetate
DM: diabetes mellitus
GP: glucose and palmitic acid
HAMSCs: human amnion‐derived mesenchymal stem cells
HBMSCs: human bone marrow mesenchymal stem cells
MAPK: mitogen‐activated protein kinase
OCN: osteocalcin
ROS: reactive oxygen species
RUNX2: runt‐related transcription factor 2
VEGF: vascular endothelial growth factor

Periodontitis is a group of inflammatory processes, which not only increases the difficulty to reconstruct oral function but also is spontaneous if left untreated 1, 2. Diabetes mellitus (DM) is established as a promoting factor for periodontitis, which aggravates the inflammation process and accelerates bone absorption 3, 4, 5. DM‐induced glucolipotoxicity upsets the balance between osteoblastic/osteoclastic differentiation and induces decreased bone mineral density 6. The skeletal phenotype of DM is characterized by osteoporosis 7, increased risk of fractures 8, poor prognosis, and inadequate bone regeneration 9. In addition, epidemiological studies suggest a biochemical link between DM and oxidative stress 10, 11. Glucolipotoxicity induces complications of DM by upregulating cell‐level stress, increasing reactive oxygen species (ROS) production, and enhancing the expression of cytokines such as tumor necrosis factor 12, 13, 14. Abnormal cellular glucose fluctuations also contribute to increased sensitivity to oxidative stress, which has a direct effect on bone resorption 15, 16.

Several approaches, including hormone therapy 17, bisphosphate 18, vitamin D 19, and tetracycline 20, have been used for treating diabetic bone defects and osteoporosis. However, these approaches are associated with various disadvantages, such as significant individual differences and limited availability. Recently, mesenchymal stem cells have been used for advancing bone engineering due to their excellent osteogenic potential 21. Previous studies confirmed that human amnion‐derived mesenchymal stem cells (HAMSCs) were capable of not only promoting human bone marrow mesenchymal stem cell (HBMSC) osteogenesis but also driving osteogenic differentiation against oxidative stress and inflammatory conditions 22, 23, 24. Therefore, it is speculated that HAMSCs have a role in DM‐induced bone deficiency.

Previous studies have shown that the p38 mitogen‐activated protein kinase (MAPK) pathway mediates anti‐osteoporosis and anti‐insulin resistance in diabetic bone deficiency 25. P38 MAPK, involved in inflammatory reaction, cell cycle regulation, and cell apoptosis, is found to be activated in the initial stage of osteoblast differentiation 26. This study further investigated the effect of HAMSCs on the p38 MAPK signaling pathway in glucose and palmitic acid (GP)‐induced HBMSCs to probe the underlying mechanism.

In this study, a transwell co‐culture system was used to investigate the effect of HAMSCs on osteoblast differentiation in GP‐induced HBMSCs. It found that HAMSCs promoted the proliferation level, increased alkaline phosphatase activity (ALP), enhanced the expression of osteoblast marker proteins, stimulated the deposition of mineralized matrix, and activated p38 MAPK. Moreover, glucolipotoxicity‐induced oxidative stress was suppressed, and vascular endothelial growth factor (VEGF) secretion was promoted by HAMSCs. These findings indicated the potential role of HAMSCs in promoting the activity of HBMSCs against glucolipotoxicity.

## Materials and methods

### Cell isolation and culture

The HBMSC cell lines were obtained from the American Type Culture Collection (PTA‐1058; ATCC, Manassas, VA, USA). Placenta was obtained from normal pregnant women (38–41 weeks) with informed consent and approval from the ethics committee of the School of Stomatology, Nanjing Medical University, China (NO.PJ2013‐037‐001). The informed consent received from patients was written, and the study methodologies conformed to the standards set by the Declaration of Helsinki. The separation of HAMSCs was performed as previously described 27. Two kinds of cells were cultured in Dulbecco's modified Eagle's medium (HyClone Laboratories Inc., Logan, UT, USA) supplemented with 10% fetal bovine serum (Gibco; Thermo Fisher Scientific, Inc., Waltham, MA, USA), 100 U·L^−1^ penicillin, and 100 mg·L^−1^ streptomycin (both Gibco; Thermo Fisher Scientific, Inc.) in a humidified atmosphere of 5% CO_2_ at 37 °C. The confluent cells were transferred to the next passage using 0.25% trypsin for up to three passages, and the culture medium was changed every 3 days.

### Co‐culture system

This study explored the effects of HAMSCs on HBMSCs in GP environments using a transwell co‐culture system (Millipore, six‐well Millicell Hanging Cell Culture Inserts, 0.4 μm, PET, Billerica, MA, USA). HBMSCs were seeded at an initial density of 5 × 10^4 ^cells·cm^−2^ in six‐well culture plates. Transwells were placed on another six‐well culture plate and seeded at increasing HBMSC : HAMSC ratios (5 × 10^4 ^cells/transwell, 10 × 10^4 ^cells/transwell, and 15 × 10^4 ^cells/transwell). After approximately 12 h, the HBMSCs were replaced with a full‐culture medium with 30 mm glucose and 100 μm palmitic acid for 24 h to induce glucolipotoxicity as previously described 28. After washing with PBS, transwells containing HAMSCs were transferred into the corresponding wells of the six‐well culture plate containing HBMSCs to create the HAMSC/HBMSC transwell co‐culture system. HBMSCs in wells with transwells served as the intervention groups, whereas HBMSCs without transwells were used as the control and model groups.

### Analysis of cellular proliferation

The proliferation level of HBMSCs was determined using flow cytometry on day 3 as described previously 29. DNA content was measured using a FACScan flow cytometer (BD Biosciences, Franklin Lakes, NJ, USA), and the cell cycle fractions (G0, G1, S, and G2 M phases) were processed using cellquest pro software (BD Biosciences). Data were analyzed using modfit lt 3.2 (Verity Software House, Topsham, ME, USA).

### *In vitro* osteogenic differentiation

When HBMSCs were treated with GP for 24 h, transwells with HAMSCs were moved to the corresponding six‐well culture plates. The cells were incubated with osteogenic medium (100 nm dexamethasone, 50 mg·mL^−1^ ascorbic acid, and 5 mm β‐glycerophosphate; Sigma Chemical Co., St. Louis, MO, USA) for 14–21 days. Cells cultures were grown in a humidified, 5% CO_2_ incubator at 37 °C. The osteogenic medium was changed every 3 days.

### Alizarin red staining

Calcium deposition of HBMSCs was determined using Alizarin red S staining after 21 days of osteogenic induction. HBMSCs were fixed for 40 min in 4% paraformaldehyde fluid at room temperature, washed twice with PBS, and then stained with 40 mm Alizarin red S staining at pH 4.2 for 20 min with gentle agitation. Mineralized nodules were visualized using an inverted microscope (Carl Zeiss AG, Oberkochen, Germany) and measured by image‐pro plus (ipp; Media Cybernetics, Manassas, VA, USA) analysis. Ten images were captured for each group, and the mean percentage was calculated.

### Analysis of alkaline phosphatase activity

On day 14, ALP activity was measured using an ALP assay kit (Jiancheng Corp, Nanjing, China) according to the manufacturer's protocols. The enzyme activity was expressed as micromoles of reaction product per minute per total protein.

### VEGF quantification

The culture supernatant of control and treatment groups was collected after 14 days and assayed to measure the level of VEGF. A human VEGF ELISA kit (R&D Systems, Minneapolis, MN, USA) was used to quantify VEGF in the medium from each group according to the manufacturer's protocols. The measured values were expressed as fold changes over that of the control: HBMSCs treated without HAMSCs.

### Osteoblast‐related protein evaluation

At the end of 14 days of co‐culture, the transwell containing HAMSCs was removed. HBMSCs were lysed in RIPA buffer (Sigma) containing protease inhibitors. Equal amounts of proteins were loaded, separated on 10% sodium dodecyl sulfate–polyacrylamide electrophoresis gels, and transferred onto polyvinylidene fluoride membranes. The membranes were blocked by incubation with 5% bovine serum albumin in PBST buffer at room temperature for 1 h and then probed overnight at 4 °C with the following monoclonal primary antibodies: calf‐related transcription factor 2 (RUNX2) antibody (1 : 1000; Cell Signaling Technology, Danvers, MA, USA #12556), osteocalcin (OCN) antibody (1 : 1000; Abcam, Cambrigenshire, UK #ab133612), p38 antibody (1 : 1000; Abcam #ab76956), and β‐actin (1 : 1000; Cell Signaling Technology #12556). Then, the membranes were incubated with horseradish peroxide‐conjugated secondary antibodies (1 : 5000; Abcam) at room temperature for 1 h. β‐Actin was used as loading control. The bands were visualized using SuperSignal West Pico Chemiluminescent Substrate (Thermo Fisher Scientific) and Kodak X‐ray film (Amersham Pharmacia Biotech, Piscataway, NJ, USA).

### ROS generation analysis

The intracellular ROS production was measured by flow cytometry and immunofluorescence staining using the substrate 2,7‐dichlorodihydrofluorescein diacetate (DCFH‐DA; Sigma), which was oxidized by ROS to 2,7‐dichlorodihydrofluorescein (DCF). After co‐culturing with HAMSCs for 48 h, HBMSCs were washed twice with PBS and incubated with DCFH‐DA (10 mm) for 30 min at 37 °C. The intensity of DCF fluorescence was determined by flow cytometry (BD Biosciences), and macrographs of DCFDA fluorescence were recorded using an inverted microscope (Carl Zeiss AG).

### Statistical analysis

If not stated differently, all data were expressed as the mean ± standard deviation from at least three independent experiments. Data were analyzed using one‐way analysis of variance followed by the Bonferroni *t*‐test for multiple comparisons. A value of *P* <0.05 was considered statistically significant.

## Results

### HAMSCs promoted the proliferation of GP‐induced HBMSCs

**Figure 1:**
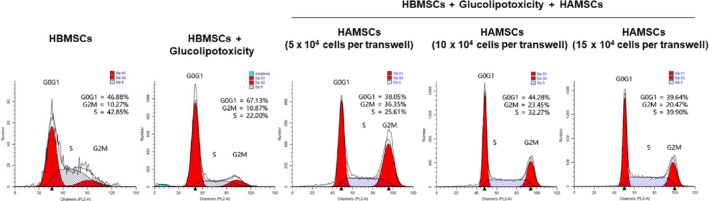
Cell cycle fractions (G0, G1, S, and G2 M phases) of HBMSCs were determined by flow cytometry on day 3.

The proliferation of HBMSCs was detected by flow cytometry on day 3. GP inhibited HBMSCs proliferation. However, the percentage of S phase improved after co‐culturing with HAMSCs and positively correlated with the HAMSC : HBMSC ratio (Fig. 1). Previous studies showed that HAMSCs promoted the proliferation of HBMSCs 30. The findings validated that HAMSCs could enhance the proliferation of GP‐induced HBMSCs.

### HAMSCs promoted mineralized matrix formation and ALP activity of GP‐induced HBMSCs

**Figure 2:**
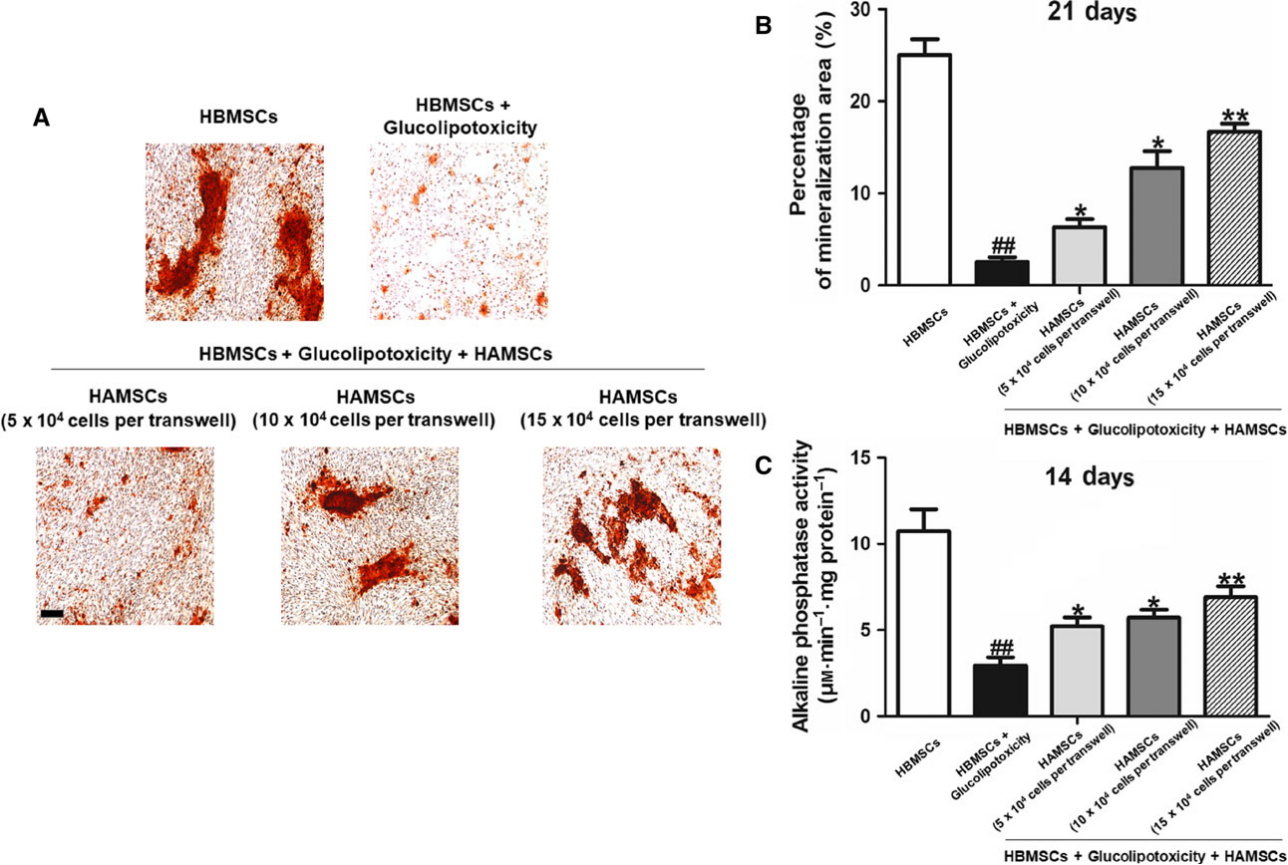
(A) Mineralized matrix deposition in HBMSCs was measured using Alizarin red S staining on day 21. (B) Mineralized nodules were visualized using an inverted microscope and measured using ipp on day 21. (C) ALP activity was measured using the ALP assay kit on day 14. The statistical significance between groups was calculated using Student's *t*‐test or ANOVA analysis as indicated. The data are expressed as the mean ± SD of three independent experiments, *n* = 3; ^##^ *P *<* *0.01 versus the HBMSC group; *P <* *0.05 and **P <* *0.01 compared with the group treated with GP alone. Scale bar: 100 μm.

Figure 2A shows the positive ratio of Alizarin red staining in the extracellular matrix of HBMSCs after 21 days. Compared with the normal cultured HBMSCs, GP reduced the formation of mineralized matrix. When co‐cultured with HAMSCs, the percentage of mineralization was upregulated (Fig. 2B). These observations indicated that HAMSCs had an active regulatory effect on the *in vitro* GP‐induced mineralization of HBMSCs.

The ALP activity of HBMSCs was measured after 14 days. GP reduced ALP activity of HBMSCs. The experimental findings indicated that HAMSCs significantly enhanced the ALP activity of HBMSCs, and ALP activity increased gradually with the HAMSC : HBMSC ratio (Fig. 2C). These results showed that HAMSCs had a potential effect on osteogenic differentiation in GP‐induced HBMSCs.

### HAMSCs promoted VEGF secretion in GP‐induced HBMSCs

**Figure 3:**
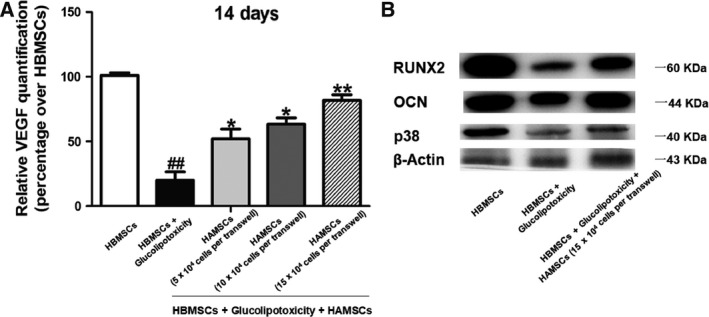
(A) VEGF level in culture supernatant from each group was measured using the VEGF ELISA kit on day 14. (B) Expression of RUNX2, OCN, and p38 proteins in GP‐induced HBMSCs cultured with or without HAMSCs after 14 days was determined by western blotting. β‐Actin served as an internal control. The statistical significance between groups was calculated using Student's *t*‐test or ANOVA analysis as indicated. The data are expressed as the mean ± SD of three independent experiments, *n* = 3; ^##^ *P *<* *0.01 versus the HBMSC group; *P <* *0.05 and **P <* *0.01 compared with the group treated with GP alone.

The positive effects of HAMSCs on GP‐induced HBMSCs were further measured on day 14. The co‐culture groups secreted much more VEGF compared with GP‐induced HBMSC groups, and the VEGF level gradually increased with the HAMSC : HBMSC ratio (Fig. 3A).

### HAMSCs promoted the expression of bone‐specific transcription factors in GP‐induced HBMSCs

The levels of OCN and RUNX2 were detected to measure the effect of HAMSCs on the osteogenesis differentiation of GP‐induced HBMSCs. The results showed that levels of RUNX2 and OCN, which were osteogenesis markers 31, 32, significantly decreased in HBMSCs of the induction groups compared with the blank control groups (*P *<* *0.05), but they improved in co‐culture groups (*P *<* *0.05). GP also inhibited the p38 in other groups compared with HBMSCs without GP treatment. However, higher levels of p38 were observed in co‐culture groups compared with those in GP‐induced single‐culture groups (Fig. 3B), suggesting the involvement of p38 MAPK in these processes.

### HAMSCs inhibited ROS production in GP‐induced HBMSCs

**Figure 4:**
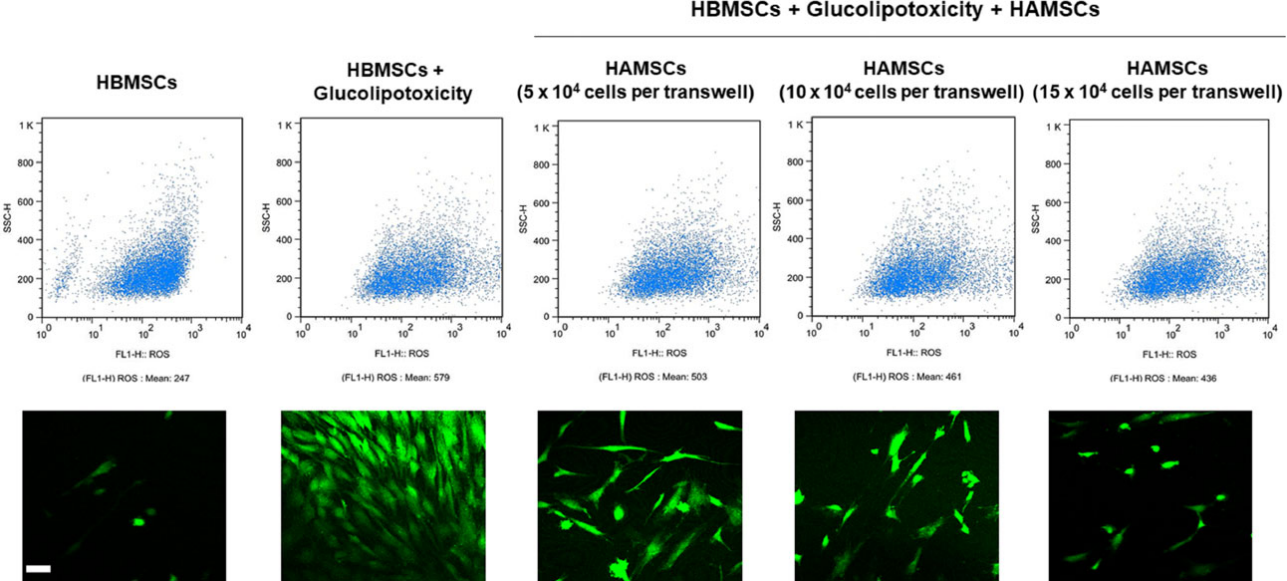
Flow cytometry and immunofluorescence staining of ROS generation in GP‐induced HBMSCs after co‐culturing with or without HAMSCs was performed after 48 h. Scale bar: 100 μm.

The ROS level in GP‐induced HBMSCs was determined to elucidate whether the beneficial effects of HAMSCs were antioxidant dependent. Figure 4 shows that GP significantly increased ROS production. However, ROS production was partially inhibited in co‐culture groups compared with GP‐induced single‐culture groups and gradually decreased with the HAMSC : HBMSC ratio. The data suggested that co‐culturing with HAMSCs inhibited the oxidative stress state of GP‐induced HBMSCs.

## Discussion

Compared with other DM complications, diabetic osteopathy has become an emerging medical and socioeconomic problem in dentistry and maxillofacial surgery 33. For example, diabetic condition accelerates the process of periodontitis and aggravates the loss of alveolar bone, seriously impairing oral function 34. High blood glucose in patients with type II diabetes strengthens osteoclast differentiation and prompts bone absorption capacity. Also, the high‐GP environment caused by diabetes leads to inhibited osteoblast differentiation and suppressed new bone formation 35.

Human amnion‐derived mesenchymal stem cells are considered to be a potential resource in bone engineering due to their beneficial characteristics 36. Previous studies showed that HAMSCs were able to reverse the bone deficiency caused by oxidative stress and inflammation 24. Consistent with the literature and previous findings, this study hypothesized the role of HAMSCs in treating the DM‐induced HBMSC injury.

In this study, HBMSCs were treated with 30 mm glucose and 100 μm palmitic acid for 24 h to form an *in vitro* injury model in diabetic bone defects. First, it found that HAMSCs promoted the proliferation of GP‐induced HBMSCs on day 3, which was confirmed by flow cytometry. Further investigations suggested that HAMSCs promoted osteogenesis in GP‐induced HBMSCs. Moreover, p38 MAPK signaling, which is a crucial trigger of osteogenic differentiation 37, was inhibited by GP treatment and reversed by HAMSCs. These results indicated a key role of HAMSCs in preventing HBMSCs injury against glucolipotoxicity through p38 MAPK.

At present, epidemiological studies suggest a biochemical link between oxidative stress and DM. Also, the role of oxidative stress in DM‐induced bone pathology has been established 38. ROS are chemical free radicals and substances that can be converted into free radicals during aerobic metabolism of cells 39. When the peripheral blood insulin resistance induced by DM starts to affect the glucose clearance rate, the continuous increase in the glycolysis flux leads to the production of intracellular ROS, resulting in potential pathological consequences 40. When the balance between oxidant/antioxidant capacity of the body is broken, oxidative stress is generated, further damaging biological molecules, cells, tissues, and organs and leading to the occurrence or development of various diseases 41. Studies have confirmed that ROS affect the function and survival of HBMSCs through a variety of mechanisms, including changes in enzyme activity, receptor signal transduction, gene expression disorder, and apoptosis 42. ROS production was assessed after 48 h by flow cytometry. The findings clearly supported the hypothesis that HAMSCs are capable of not only promoting the proliferation and osteogenic differentiation of GP‐induced HBMSCs but also inhibiting the GP‐induced oxidative stress due to their antioxidant property.

Bone needs intraosseous vasculature to maintain normal metabolism 43. VEGF, a cytokine involved in angiogenesis and osteogenesis, evidently enhanced osteogenesis 44, 45. In this study, a higher level of VEGF secretion was found in HASC/HAMSC groups, implying that HAMSCs exerted their effects via cytokines.

In conclusion, the present study highlighted the potential role of HAMSCs in promoting proliferation, driving osteogenic differentiation, and reducing oxidative stress in GP‐induced HBMSCs. These findings suggested that HAMSCs might serve as an appropriate treatment for diabetes‐related bone complications. Further investigations are warranted to fully elucidate the complete molecular mechanisms.

## Conflict of interest

The authors declare no conflict of interest.

## Author contributions

YB performed the experiments and wrote the original draft; XM interpreted the data; RW and HY performed some experiments; NC and YD supervised the study, designed the experiments, and wrote the manuscript.
